# Bioinformatics-Based Analysis of lncRNA-mRNA Interaction Network of Mild Hepatic Encephalopathy in Cirrhosis

**DOI:** 10.1155/2021/7777699

**Published:** 2021-12-13

**Authors:** Ke Wang, Yanzhen Lu, Zhifeng Zhao, Chihao Zhang

**Affiliations:** ^1^Department of Hepatobiliary and Pancreatic Surgery, Ningbo Medical Treatment Centre Li Huili Hospital, Ningbo University, Ningbo, Zhejiang, China; ^2^Department of Gynaecology, Beilun People's Hospital, Ningbo, Zhejiang, China; ^3^Department of General Surgery, Shanghai Ninth People's Hospital, Shanghai Jiao Tong University School of Medicine, Shanghai, China

## Abstract

**Backgrounds:**

Serum long noncoding RNAs (lncRNAs) and messenger RNAs (mRNAs) interaction network was discovered to exert an important role in liver cirrhosis while little is known in mild hepatic encephalopathy (MHE). Therefore, we aim to systematically evaluate the serum lncRNA-mRNA network and its regulatory mechanism in MHE.

**Methods:**

The data of serum mRNAs and lncRNAs were derived from the Gene Expression Omnibus (GEO) database. The differentially expressed genes (DEGs) were calculated between 11 cirrhotic patients with and without MHE. Next, the biological functions and underlined pathways of DEGs were determined through Gene Ontology (GO) and Kyoto Encyclopedia of Genes and Genomes (KEGG) pathway analyses. Finally, an interactive network between lncRNAs and mRNAs was built, and hub genes were identified, respectively.

**Results:**

A total of 64 differentially expressed lncRNAs (dif-lncRNAs) were found between patients with and without MHE, including 30 up- and 34 downregulated genes. 187 differentially expressed mRNAs (dif-mRNAs) were identified, including 84 up- and 103 downregulated genes. Functional enrichment analysis suggested that the regulatory pathways involved in MHE mainly consisted of a series of immune and inflammatory responses. Several hub mRNAs involved in regulatory network were identified, including CCL5, CCR5, CXCR3, CD274, STAT1, CXCR6, and EOMES. In addition, lnc-FAM84B-8 and lnc-SAMD3-1 were found to regulate these above hub genes through building a lncRNA-mRNA network.

**Conclusion:**

This is the first study to construct the serum lncRNA-mRNA network in MHE, demonstrating the critical role of lncRNAs in regulating inflammatory and immunological profiles in the developing of MHE, suggesting a latent mechanism in this pathophysiological process.

## 1. Background

Mild hepatic encephalopathy (MHE) is a neuropsychiatric complication of severe liver cirrhosis accompanied by attention deficit, mild cognitive impairment, intellectual disability, and coordination disorders [1]. It was reported that cirrhotic patients have 20%–80% of risk of developing MHE [2]. Ammonia remains a critical role in the pathophysiology of MHE development. But recently, various novel mechanisms were identified, involving the alterations of gut microbiome, neuroinflammation, and neuroimmune axis [3]. Those pathways constitute a sophisticated network and contribute to the formation and development of MHE. However, little is known about these pathways' regulatory networks and their interconnections.

Gene microarrays provide a large amount of analyzable data, which made it widely used in brain disorders [4]. At present, numerous multiomic database of brain metabolism including MHE already existed in the Gene Expression Omnibus (GEO) database [5, 6]. In those datasets, diverse biological specimens from the brain tissue, serum, and other body fluids are detected, which allow the modeling of a highly complex metabolic network and the identification of specific features of MHE. In a recent study, Rubio et al. [6] profile the serum transcriptome in MHE patients and discovered that altered immune pathways may be critical for the development of MHE.

Long noncoding RNA (lncRNA), with length more than 200 nucleotides, plays an important role by regulating the gene expression. Those lncRNAs do not encode proteins but exert their action by interfering gene transcription involving inflammation, apoptosis, and oxidative stress [7–10]. At present, cirrhosis have been confirmed to be regulated by numerous lncRNAs, such as GAS5 [11], H19RNA [12], lnc-LFAR1 [13], and PVT1 [14]. In HE induced by acute liver failure, the function of cerebral lncRNAs has also been documented to be related to inflammation, neuropathology, etc. [15]. However, there is still no systematic and comprehensive analysis concerning the role of lncRNA regulatory network in the occurrence of MHE in liver cirrhosis.

In this study, serum transcriptome data of cirrhotic patients were obtained from the GEO database (GSE149741). Firstly, the differentially expressed genes (DEGs) of MHE were identified including differentially expressed lncRNAs (dif-lncRNAs) and differentially expressed mRNAs (dif-mRNAs). Then, the pathophysiological processes regulated by dif-lncRNAs were analyzed through Gene Ontology (GO) function annotation and Kyoto Encyclopedia of Genes and Genomes (KEGG) enrichment. Finally, the core lncRNAs were identified and their regulatory network with hub mRNAs was explored by constructing mRNA-lncRNA interaction network.

## 2. Method

### 2.1. Data Collection

The original microarray dataset (GSE149741) was downloaded from the GEO database (https://www.ncbi.nlm.nih.gov/geo/), including 5 cases without MHE and 6 cases with MHE [6]. The datasets were reannotated and divided into mRNA and lncRNA files before analysis [16]. The quantile normalization of gene expression matrix was conducted using normalizeBetweenArrays feature of *limma* package (v. 3.40.6) of R (v. 4.0.4).

### 2.2. Differential Gene Expression Analysis

The identification of differential gene expression was carried out using the *limma* package [17]. *P* < 0.05 and ∣log2 (fold change) | >1.5 were used as cut-off criteria. Heatmaps on dif-lncRNAs and dif-mRNAs were drawn using *pheatmap R* package. Volcano plots were generated using *ggplot2 R* package.

### 2.3. GO Function and KEGG Pathway Analyses

GO function and KEGG pathway analyses of dif-lncRNAs were conducted by using *clusterProfiler* package [18]. The GO terms define the concepts of gene function and those interactive relationships which consisted of biological processes, molecular functions, and cellular components. KEGG analysis illustrated the gene functions and biological pathways. The adjusted *P* < 0.05 was regarded as cut-off criteria [19, 20].

### 2.4. Protein–Protein Interactions (PPI) and Hub Genes

The STRING database (https://string-db.org/) is a database for interactive gene search to construct the protein–protein interactive network [21]. An interaction with a combined score > 0.4 was recognized as statistically significant. Based on the STRING database, Cytoscape (version 3.6.1) was employed to visualize the mRNA interaction network [22]. The most important module was identified by Molecular Complex Detection (MCODE, version 1.4.2), a plug-in of Cytoscape [23]. In order to evaluate the hub genes, centrality of the mRNA node was calculated using the CytoHubba, a plug-in of Cytoscape [24]. The ‘MCC' method in CytoHubba was employed to identify the featured nodes and hub genes. The node level is quantified by summarizing the local topology of the connection numbers [25]. The top 10 genes ranked by their centrality degree were considered as hub mRNA.

### 2.5. lncRNA-mRNA Network Construction

The paired lncRNA-mRNA coexpression data was analyzed to generate the regulatory network. Hub lncRNAs were identified via this network. Subsequently, the Pearson correlation analysis between hub lncRNAs and regulated mRNAs was conducted. The absolute value correlation coefficient > 0.8 with *P* < 0.05 was considered a strong correlation.

## 3. Results

### 3.1. Quality Control and Preprocessing

**Figure 1:**
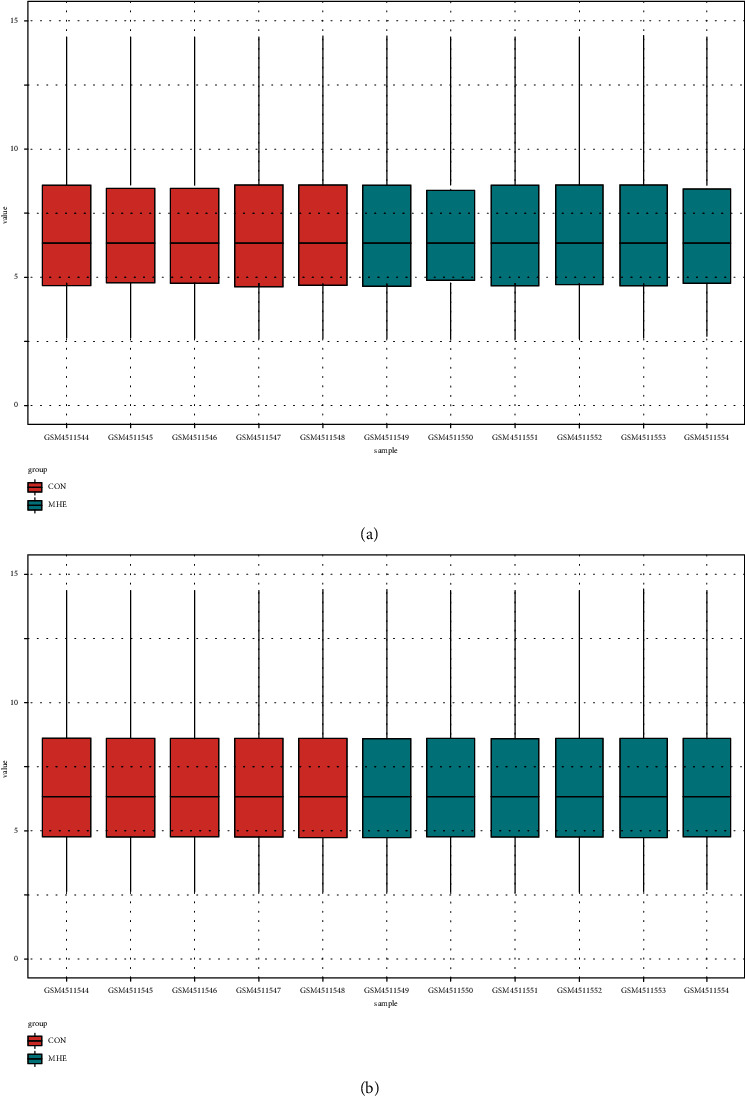
Normalization of GSE149741 data. (a) Data before normalization and (b) data after normalization.

The microarray dataset was derived from a total of 11 cirrhotic patients, including 6 with MHE and 5 without MHE. After quality control, the intensity distribution of the original data was normalized to eliminate differences between individuals and the baselines of each sample remained basically the same (Figure 1).

### 3.2. Differential Expression Analysis of lncRNAs and mRNAs

**Figure 2:**
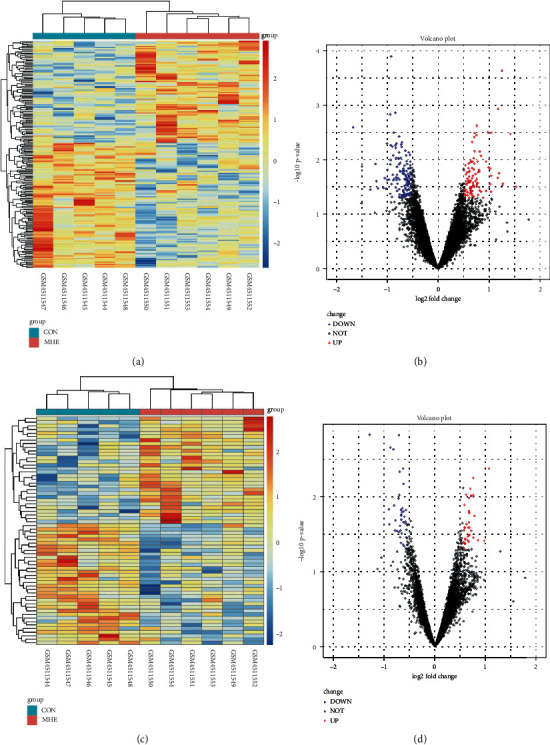
Heatmaps of differentially expressed analysis of mRNAs and lncRNAs. (a) Heatmaps of differentially expressed mRNAs. (b) Volcano plot of differentially expressed mRNAs. (c) Heatmaps of differentially expressed lncRNAs. (d) Volcano plot of differentially expressed lncRNAs.

From the lncRNA and mRNA expression profiles, a total of 64 dif-lncRNAs were identified among which 30 lncRNAs were upregulated and the rest were downregulated (Figures 2(a) and 2(b)). Meanwhile, 187 dif-mRNAs were found to be differentially expressed with 84 upregulated and 103 downregulated genes (Figures 2(c) and 2(d)). Then, the clustering heat map (Figures 2(a) and 2(c)) and volcano map (Figures 2(b) and 2(d)) were used to visualize the different expressions of lncRNAs and mRNA between two groups. From the figure, it can be drawn that the DEG in the MHE group was significantly separated from those without MHE, suggesting that these DEGs could distinguish MHE patients from the others.

### 3.3. GO and KEGG Pathway Analysis of lncRNA Targeted Genes

**Figure 3:**
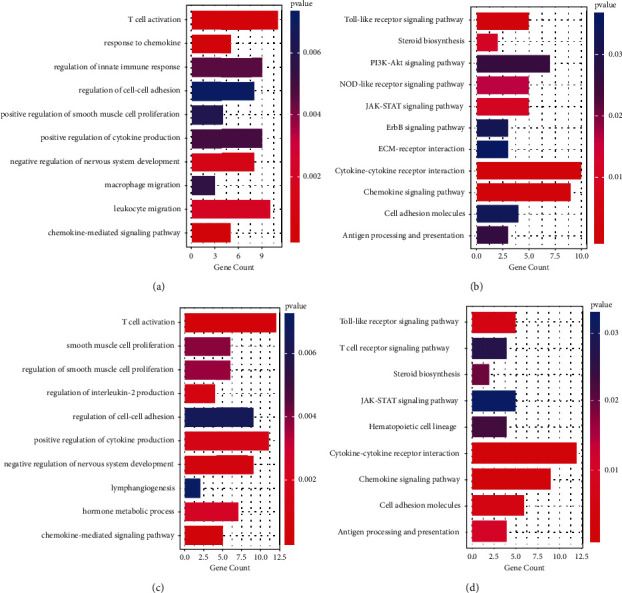
Analysis of Gene Ontology biological process (GO-BP) and KEGG pathways. (a) Top GO-BP pathways of upregulated lncRNAs. (b) Top enriched KEGG analysis of upregulated lncRNAs. (c) Top GO-BP pathways of downregulated lncRNAs. (d) Top enriched KEGG analysis of downregulated lncRNAs.

In GO analysis, upregulated lncRNAs were significantly related to T cell activation, response to chemokines, innate immune response, cell adhesion, smooth muscle cell proliferation, cytokines, macrophages, and leukocyte migration (Figure 3(a)). The downregulated lncRNAs were mainly enriched in T cell activation, smooth muscle cell proliferation, smooth muscle cell proliferation regulation, interleukin-2 production regulation, intercellular adhesion regulation, positive regulation of cytokine production, negative regulation of nervous system development, lymphangiogenesis, hormone metabolism process, and chemokine-mediated signal pathway (Figure 3(c)).

In KEGG analysis, the enrichment pathways for upregulated lncRNAs included Toll-like receptor signaling pathway, steroid biosynthesis, Pl3K-akt signaling pathway, Nod-like receptor signaling pathway, JAK-STAT signaling pathway, ErbB signaling pathway, ECM-receptor interaction, cytokine-cytokine receptor interaction, chemokine signaling pathway, cell adhesion molecules, and antigen processing and presentation(Figure 3(b)). All these pathways are related to the progression of liver cirrhosis and hepatic encephalopathy [26, 27]. The enrichment pathways for downregulated lncRNAs included Toll-like receptor signaling pathway, T cell receptor signaling pathway, steroid biosynthesis, JAK-STAT signaling pathway, hematopoietic cell lineage, cytokine-cytokine receptor interaction, chemokines signal pathways, cell adhesion molecules, and antigen processing and presentation (Figure 3(d)).

### 3.4. PPI Network and Identification of Hub mRNAs

**Figure 4:**
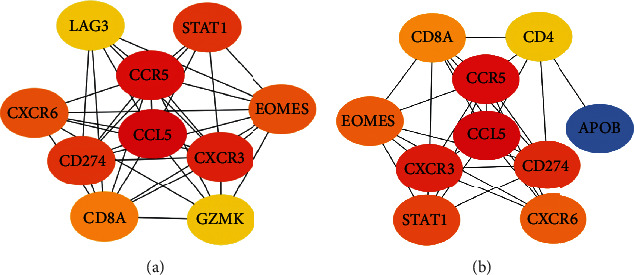
Identification of hub mRNAs in PPI network regulated by dif-lncRNAs. (a) Top 10 hub mRNAs in the PPI network of the upregulated genes. (b) Top 10 hub mRNAs in the PPI network of the downregulated genes.

The CytoHubba in Cytoscape was used to analyze the STRING data and construct the PPI network regulated by dif-lncRNAs. Using the MCC method, the hub mRNAs in this network were identified, in which upregulated genes including CCL5, CCR5, CXCR3, CD274, STAT1, CXCR6, EOMES, CD8A, GZMK, and LAG3 (Figure 4(a)). The downregulated genes including CCL5, CCR5, CXCR3, CD274, STAT1, CXCR6, EOMES, APOB, CD8A, and CD4 (Figure 4(b)). These mRNAs have high betweenness centrality (BC value), suggesting that they may be potential key regulatory factors.

### 3.5. lncRNA-mRNA Regulatory Network and Identification of Hub lncRNAs

**Figure 5:**
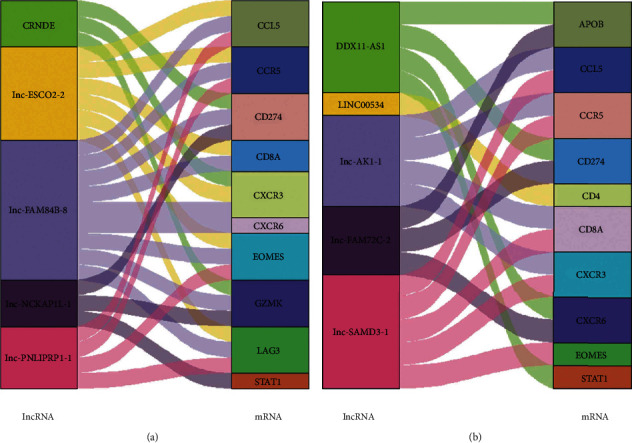
Identification of hub lncRNAs based on hub mRNAs through lncRNA-mRNA regulatory network. (a) Top lncRNAs in upregulated dif-lncRNA-associated network. (b) Top lncRNAs in downregulated dif-lncRNA-associated network.

Based on hub mRNAs identified above, the hub lncRNAs were investigated through the lncRNA-mRNA regulatory network. As a result, several hub lncRNAs were identified, including lnc-FAM84B-8, lnc-ESCO2-2, lnc-PNLIPRP1-1, lnc-NCKAP1L-1, and CRNDE in upregulated dif-lncRNA-associated network (Figure 5(a)) and lnc-SAMD3-1, lnc-AK1-1, DDX11-AS1, lnc-FAM72C-2, and LINC00534 in downregulated dif-lncRNA-associated network (Figure 5(b)). Among those hub lncRNAs, lnc-FAM84B-8 and lnc-SAMD3-1 were identified as the core lncRNAs as they were most closely related to the hub mRNA network. We can find that the expression of lnc-FAM84B-8 increased in HE, while lnc-SAMD3-1 decreased when patients developed into HE (Supplementary Fig. 1). The two core lncRNAs above represented the upregulated and downregulated lncRNAs, respectively.

### 3.6. Correlation Analysis between Core lncRNAs and mRNAs

**Figure 6:**
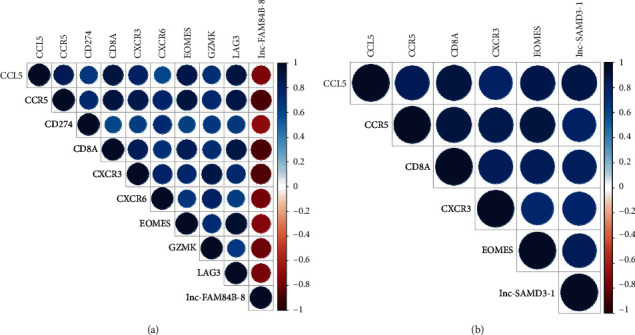
Correlation analysis between core lncRNAs and mRNAs. (a) Correlation analysis between lnc-FAM84B-8 as core lncRNA and all the hub mRNAs associated with upregulated dif-lncRNAs. (b) Correlation analysis between lnc-SAMD3-1 as core lncRNA and all the hub mRNAs associated with downregulated dif-lncRNAs.

The Pearson correlation coefficients were assessed between core lncRNAs above and hub mRNAs. The results revealed that there was a significant negative correlation between lnc-FAM84B-8 and all the upregulated dif-lncRNA-associated hub mRNAs (Figure 6(a)). At the same time, a significant positive correlation was also verified between lnc-SAMD3-1 and all the downregulated dif-lncRNA-associated hub mRNAs (Figure 6(b)). The results suggested that the expression profiles of core lncRNAs and hub mRNAs were significantly correlated.

## 4. Discussion

In this study, serum lncRNAs-mRNAs interactive network was built in cirrhosis-related MHE patients. The enrichment analysis indicated that the lncRNA regulatory genes were mostly associated with inflammation, innate, and adaptive immune response. The core lncRNAs including lnc-FAM84B-8 and lnc-SAMD3-1 were furtherly identified in up-/downregulated lncRNA-associated gene network separately. The Pearson correlation analysis implied that two lncRNAs were associated with the hub mRNAs strongly, which hinted the crucial role of these two lncRNAs in the progression of MHE. As far as we know, this is the first study to investigate the serum lncRNAs in cirrhosis-related MHE.

Silva et al. [15] priorly examined the cerebral lncRNAs in HE associated with acute liver failure and discovered that the lncRNAs are mainly involved in inflammation and neuropathology. Rubio et al. [6] furtherly studied the peripheral gene expression network in cirrhotic patients with MHE systemically and found that immune systems occupy the core status. In this study, several upregulated cytokines were identified including CCL20, CX3CL1, CXCL13, IL-15, IL-22, and IL-6, involving 3 immune-related pathways, including “chemotaxis,” “adaptive immune response,” and “immune response.” As mentioned above, the inflammatory and immune responses exert an essential role in HE, and the significance of cerebral lncRNAs was also investigated in acute liver failure-associated HE. However, the systemic regulatory functions of serum lncRNAs in cirrhosis-related MHE still remain unknown. Hereby, our study filled the gap and summarized the peripheral lncRNA-mRNA network systemically in the occurrence and development of cirrhosis-related MHE. In our study, the lncRNA regulated network mainly consisted of immune and inflammatory systems and play an important role in the development of cirrhosis-associated MHE, which were consistent with the previous studies.

As far as we know, liver fibrosis is often accompanied by systemic inflammatory responses, which could influence the brain function and play important roles in the pathogenesis of HE [28]. In this process, numerous lncRNAs have been implicated in different stages, with involvement of variable immune inflammatory responses. For example, lncRNA lnc-JAM2-6 has been found to participate in the inflammatory response in nonalcoholic fatty liver disease (NAFLD), a precursor of liver fibrosis [29]. In liver fibrosis, numerous lncRNAs such as MALAT1, LFAR1, H19, and NEAT1 was implicated in various inflammatory chemokine pathways including transforming growth factor *β* (TGF-*β*), activation of macrophage, and C-X-C motif chemokine ligand 5 (CXCL5) [30–33]. Additionally, our research also confirmed the regulating effects of lncRNAs on immunological inflammation in cirrhosis-related MHE.

As a common complication of cirrhosis, HE is largely influenced and deteriorated by alterations of the immune and inflammatory systems. D'Mello et al. discovered that the increase of circulating inflammatory factors induced by liver injury altered the neurological function in mice significantly [34]. Görg et al. investigated the gene expressions in brain samples of HE patients and found upregulated genes associated with oxidative stress, microglia activation, inflammatory pathways, cell proliferation, and apoptosis [35]. In another study, we confirmed the effect of immune and inflammation responses in bile duct ligation (BDL) HE rat model [36]. In this study, neuroinflammatory reaction in the brain was found aggravated significantly in the BDL rats' cortex, which was verified by the high expression inflammatory factors such as interleukin 1*β*, interferon *γ*, tumor necrosis factor *α*, and ionized calcium binding adaptor molecule 1 (Iba1). In addition, as an important event of neuroinflammation, microglial activation was also detected in BDL rats. To ameliorate the inflammation, we tried to stimulate meningeal lymphangiogenesis by injecting adeno-associated virus–vascular endothelial growth factor C (AAV-VEGF-C), a potent lymphangiogenesis stimulatory factor, and we were surprised to find that the expression of those inflammatory factors was decreased by increasing lymph efflux and the motor function of the rats were recovered in some extent. Besides, we also found that the level of ammonia in the brain tissue of BDL rats did not increase significantly, suggesting that the inflammation may play a dominant role in the development of HE. In our study, the regulatory functions of lncRNAs in MHE were also found related to immune inflammatory processes. Considering trigger role of inflammation and immunophenotype in the development of MHE, lncRNAs might be a crucial component in this process with its immunoregulatory functions.

The lncRNAs-related pathways discovered in the enrichment analysis were mainly consisted of immune and inflammatory factors, which were implicated in HE according to preceding studies, including Toll-like receptors, steroid, cytokines, chemokines, cell adhesion molecules, AKT/JAK-STAT pathways, and antigen processing and presentation. For example, Jayakumar et al. [37] discovered that cerebral edema in acute HE was induced by elevated Toll-like receptor 4 expression in brain endothelial cells. Butterworth [38] uncovered that cerebral neurosteroids increased significantly in the brains of HE patients. In addition, lipid metabolism has also been defined as related to MHE [6]. As for MHE-related cytokines, a variety of cases were identified including IL-1*β*, IL-6, IFN*γ*, IL-17*α*, IFN*λ*2, and IFN*λ*3 [39]. Furthermore, the severity of HE is also closely associated to the level of cytokines, especially TNF-*α* [40]. Regarding chemokines, neuron CCL2 is upregulated and participates in microglia activation and neurological decline during HE [41, 42]. Soluble nerve cell adhesion molecule (sNCAM) was also reported to be an important risk factor for HE in HCC patients [43]. Moreover, AKT [44] and JAK-STAT [45] showed significant changes within HE. Those studies above provided possible explanations for the immune inflammatory responses involved in the HE development.

This study still has certain limitations. First of all, only GEO database data was employed in our analysis, which lacked clinical samples to confirm. Meanwhile, the mechanism of regulatory network in MHE also needs to be further explored. In future investigations, rat MHE models should also be constructed in order to fortify the related molecular pathway mechanisms.

## 5. Conclusion

In summary, based on bioinformatics analysis, our study showed that the serum lncRNAs-mRNA interaction network may play a critical role in cirrhosis-associated MHE by regulating the inflammatory and immunological systems, implying a latent mechanism in the pathophysiological process of MHE. Additionally, two core lncRNAs were identified including lnc-FAM84B-8 and lnc-SAMD3-1, which may serve as key targets for intervention in MHE. Additional research is required to demonstrate the therapeutic effectiveness of these lncRNAs.

## Data Availability

Please contact the author for data requests.

## Abbreviations

AAV-VEGF-C: Adeno-associated virus–vascular endothelial growth factor C
BDL: Bile duct ligation
CXCL5: C-X-C motif chemokine ligand 5
DEGs: Differentially expressed genes
dif-lncRNAs: Differentially expressed lncRNAs
dif-mRNAs: Differentially expressed mRNAs
GEO: Gene Expression Omnibus
GO: Gene Ontology
Iba1: Ionized calcium binding adaptor molecule 1
KEGG: Kyoto Encyclopedia of Genes and Genomes
lncRNAs: Long noncoding RNAs
mRNAs: Messenger RNAs
MHE: Mild hepatic encephalopathy
MCODE: Molecular complex detection
NAFLD: Nonalcoholic fatty liver disease
PPI: Protein–protein interactions
sNCAM: Soluble nerve cell adhesion molecule
TGF-*β*: Transforming growth factor *β*.

## Supplementary Materials

Supplementary Materials Supplementary Figure 1: the expression comparison of lnc-FAM84B-8 (a) and lnc-SAMD3-1 (b) between control and MHE group.
