# Supplementary figures and images for: Bioinformatics-Based Analysis of lncRNA-mRNA Interaction Network of Mild Hepatic Encephalopathy in Cirrhosis
Source: Comput Math Methods Med. 2021 Dec 13;2021:7777699. doi: 10.1155/2021/7777699 (PMC8687767; doi:10.1155/2021/7777699)

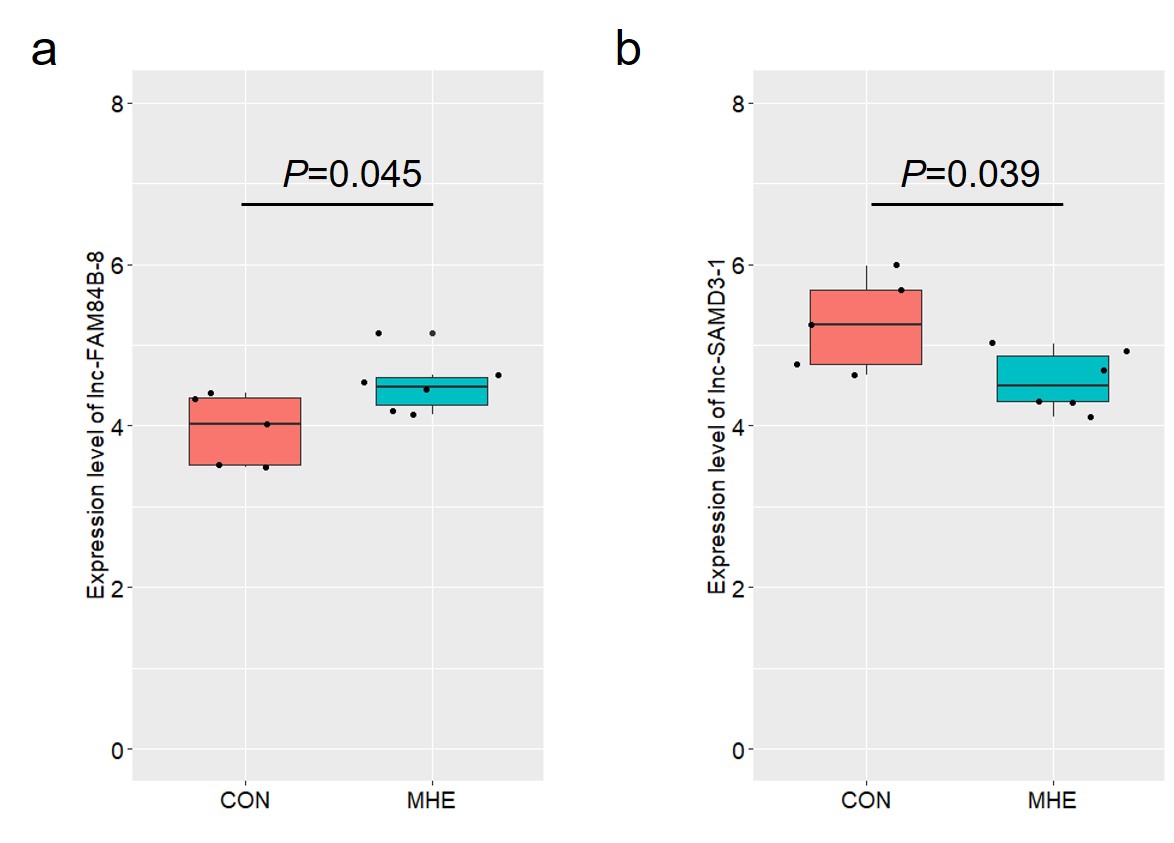

Supplement: Supplementary Materials — Supplementary Figure 1: the expression comparison of lnc-FAM84B-8 (a) and lnc-SAMD3-1 (b) between control and MHE group. [file 7777699.f1.jpg]
